# The Protective Effect of Mycosporine-Like Amino Acids (MAAs) from *Porphyra yezoensi*s in a Mouse Model of UV Irradiation-Induced Photoaging

**DOI:** 10.3390/md17080470

**Published:** 2019-08-14

**Authors:** Rui Ying, Zhaohui Zhang, Huiying Zhu, Bafang Li, Hu Hou

**Affiliations:** College of Food Science and Engineering, Ocean University of China, No.5, Yu Shan Road, Qingdao 266003, China

**Keywords:** *Porphyra yezoensis*, MAAs, antioxidant enzymes, inflammation, NF-κB

## Abstract

The objective of this research was to extract and prepare mycosporine-like amino acids (MAAs) and investigate the mechanism by which they act against UV-induced skin photoaging in Institute of Cancer Research (ICR ) mice. MAAs such as porphyra-334 and shinorine were extracted from *Porphyra yezoensis*, separated, and purified using column chromatography with SA-2 cation exchange resin. The effects of MAAs on the activity of endogenous antioxidant enzymes, namely total superoxide dismutase (T-SOD), glutathione peroxidase (GSH-Px), catalase (CAT), and malondialdehyde (MDA) were analyzed in mouse skin tissue. Pathological changes of skin tissue caused by ultraviolet radiation and the arrangement of collagen were observed by Hematoxylin-Eosin (HE) staining and scanning electron microscopy (SEM). Interleukin 1β (IL-1β), IL-6, and IL-10 were detected using the quantitative real-time reverse transcription-polymerase chain reaction (qPCR) and Enzyme Linked Immunosorbent Assay (ELISA). The concentration and expression of these proinflammatory cytokines was associated with the presence of nuclear factor (NF)-κB. The results show that MAA compounds from *Porphyra yezoensis* could suppress UV-induced photoaging of skin by inhibiting the reduction of endogenous antioxidant enzymes. Compared to the control group, the concentrations of SOD, GSH-Px, and CAT increased significantly in skin tissue homogenate following the external administration of MAAs (*p < 0.05, p < 0.01*), while the content of MDA decreased significantly (*p < 0.05*). Meanwhile, the administration of MAAs was associated with down-regulations in the concentration and mRNA expression of NF-κB, IL-1β, IL-6, and IL-10. The results suggest that MAAs could protect skin from photodamage by increasing antioxidant enzyme activities and inhibiting inflammation.

## 1. Introduction

Skin photoaging refers to cutaneous damage caused by exposure to ultraviolet radiation that leads to skin changes, such as erythema, cornification, melanin sedimentation, and skin aging [1]. When Ultraviolet (UV) radiation penetrates the atmosphere and reaches the Earth's surface, it causes different degrees of damage to the skin dermis and epidermis depending on its wavelength [2]. The effects of Ultraviolet A (UVA), (long wavelength ultraviolet) radiation cause dermal damage, skin keratinization, the breaking of collagen fibers, and melanin sedimentation. Meanwhile, Ultraviolet B (UVB ), (shorter wavelength ultraviolet) radiations mainly affects the skin’s epidermal layers, causing erythema, edema, and wrinkles [3,4]. Exposure to UV irradiation is also associated with an oxidative stress response due to the accumulation of reactive oxygen spices (ROS) [5]. 

When the presence of excess ROS results in oxidative stress, the activity of endogenous antioxidant enzymes also decrease. Consequently, the endogenous antioxidant enzyme system, including T-SOD, GSH-Px, and catalase, have significantly lower effects on skin tissue and the level of malondialdehyde (MDA) is significantly higher than under redox balance. This can lead to further UV radiation-induced oxidative damage to skin tissue and photoaging.

In UV-induced photoaged skin, ROS trigger signaling transduction and activate nuclear factor-kappa B (NF-κB), a mediator of inflammation [6]. The inflammatory immune response, which is mediated by NF-κB, is reflected by the expression of proinflammatory cytokines such as IL-1β, IL-6, and IL-10 [7,8,9] and may enhance the degree of pathological damage in photoaging skin tissue. This response oxidizes proteins and DNA, leading to oxidant damage and dermal carcinogenesis [10,11]. As a result, UV radiation-induced photo-oxidative damage causes irreversible injury to skin tissue as well as immune suppression at both the molecular and cellular levels.

Mycosporine-like amino acid compounds have been reported to have multiple types of activity, such as ultraviolet light absorption and antioxidant and anti-photoaging activity, as a result of their phenolic hydroxyl structure, which can effectively dispel reactive oxygen species (ROS) [12]. Mycosporine-like amino acids extracted from algae have strong ultraviolet absorption capacity and anti-photoaging properties [13], which provide effective protection against UV irradiation-induced photo-oxidative damage by inhibiting the expression of the matrix metalloproteinase (MMP) gene by suppressing the Mitogen Activated Protein Kinase (MAPK) signal pathway [14]. However, the effects of MAAs on the UV irradiation-induced inflammation response and the photoaging protective mechanism(s) need to be further investigated.

Previous studies confirmed that MAA compounds could guard against UV irradiation photoaging by regulating the NF-κB and MAPK signaling pathway and suppressing the expression of matrix metalloproteinases (MMPs) [12]. Based on above previous research, the objective of this study was to establish a skin photoaging mouse model that can be used to further research the mechanism by which MAAs prevent inflammation-induced damage to photoaging skin. By comparing the pathological features of skin tissue, the levels of proinflammatory cytokines and the mRNA expression, and the anti-photoaging mechanism of MAAs, the suppression of oxidative stress and the immune response were further analyzed.

## 2. Results

### 2.1. The Components of MAAs Extracted from Porphyra yezoensis 

**Figure 1:**
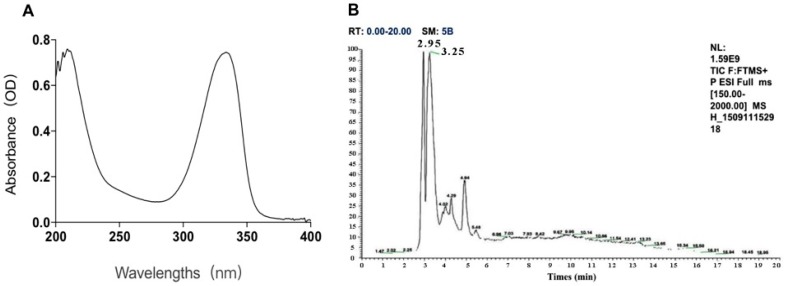
The identification of compounds from *porphyra yezoensis.* (**A**) Absorption spectra of extraction with alcohol solutions of *Porphyra yezoensis*; (**B**) Total Ion Chromatogram of Porphyra yezoensis showing the eluted compounds profile.

**Figure 2:**
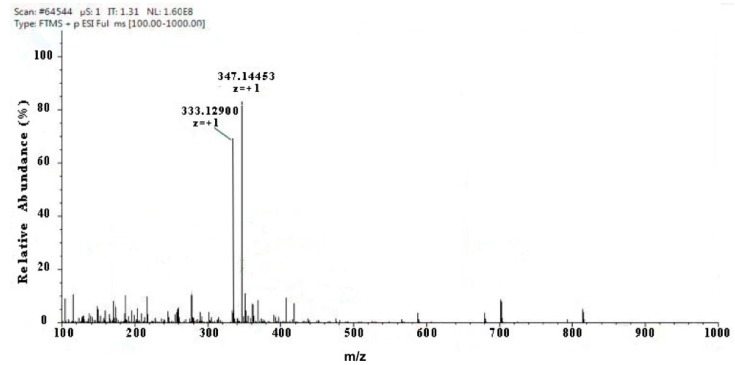
Full mass spectra showing mycosporine-like amino acid (MAA) compounds of Porphyra yezoensis eluted in 2.95 or 3.25 peak shown above in Total Ion Chromatogram (TIC).

**Figure 3:**
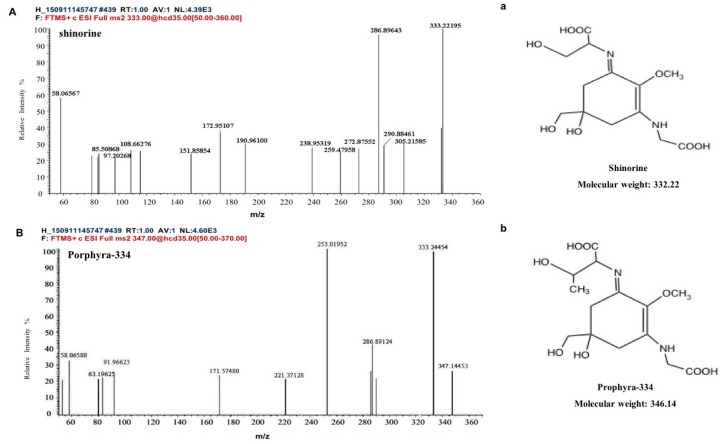
Structure elucidation for MAA compounds from *Porphyra yezoensis*. (**A**) MS/MS analysis of the shinorine; a: the structure of shinorine; (**B**) MS/MS analysis of porphyra-334; b: the structure of porphyra-334.

Two kinds of major MAA compounds were identified from *Porphyra yezoensis* using HPLC-UV-MS analysis. The compounds extracted from *Porphyra yezoensis* were characterized by UV-visible spectroscopy at 200–400 nm, which revealed the maximum UV absorption peak at 334 nm, as shown in Figure 1a. This is in accordance with the molar absorption coefficients of characteristic MAA compounds [15]. Furthermore, the total ion current chromatogram indicated that two main components were involved. Using photo array detection (PAD), retention times were identified at 2.95 and 3.25 min with two prominent peaks and the characteristic UV absorption spectra at 334 nm, as shown in Figure 1b. High-performance liquid chromatography (HPLC) combined with mass spectrometry (MS) was used to analyze and identify the two prominent MAA compounds with mass charge ratio (*m/z*) values of 333.1 and 347.1, respectively, as shown in Figure 2. By contrasting the second-order mass spectrum fragment ions peaks and *m/z*, the sample components were determined to be shinorine and porphyra-334 [15], the molecular structures of which are shown in Figure 3. Combined with molecular absorption coefficient for shinorine and porphyra-334, the composition of MAAs extracted from *Porphyra yezoensis* each component were: porphyra-334 = 0.876%, shinorine = 1.039%.

### 2.2. Effect of MAAs on Fibroblast Cytotoxicity

**Figure 4:**
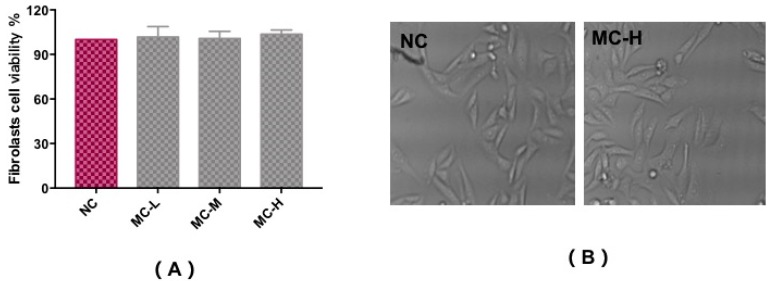
Cytotoxicity test to assess the fibroblast cell viability of MAAs (**A**) and the morphological characteristics (**B**). Note: NC group: DEME medium added; MC-L group: 0.5% MAAs and DEME medium; MC-M group: 1.0% MAAs and DEME medium; MC-H group: 2.0% MAAs and DEME medium.

In vitro cytotoxicity tests using the MTT assay were carried out to assess the fibroblast cell toxicity of MAA compounds, as shown in Figure 4A,B. Compared with a control group (NC), trypan blue staining showed that cells had a survival rate of above 90%, with a regular cell shape, and no observed cytotoxicity with different concentrations of MAA solution. This illustrates that MAA compounds could act as a kind of natural ultraviolet protection agent for ultraviolet protection.

### 2.3. Pathological Histology Analysis

**Figure 5:**
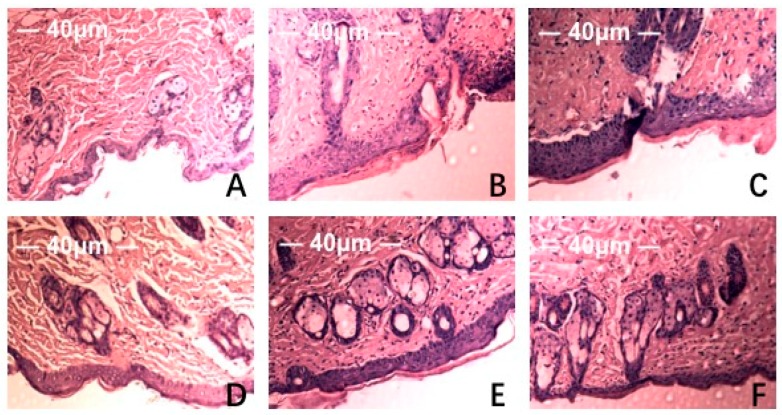
Effect of MAAs on the histological appearance of ICR mouse skin (HE staining 40×). Note: (**A**) control group; (**B**) model group, treatment with 120 μL distilled water; (**C**) model positive group, treatment with vitamin C (0.06 mg per mouse for one day); (**D**) MAAs-L group, treatment with MAAs (0.6 mg per mouse for one day); (**E**) MAAs-M group, local treatment with MAAs (1.2 mg per mouse for one day); (**F**) MAAs-H group, local treatment with MAAs (2.4 mg per mouse for one day).

**Figure 6:**
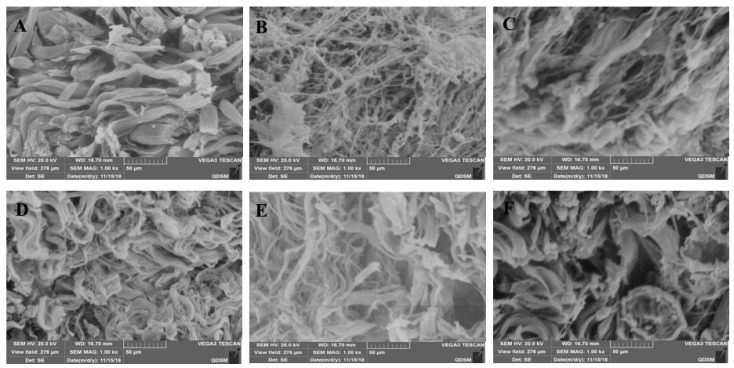
MAAs on the skin collagen fibers of the IRC mouse, as determined by scanning electron microscopy (SEM) (×1000). Note: (**A**) control group; (B) model group, treatment with 120 μL distilled water; (**C**) model positive group, treatment with vitamin C (0.06 mg per mouse for one day); (**D**) MAAs-L group, treatment with MAAs (0.6 mg per mouse for one day); (**E**) MAAs-M group, local treatment with MAAs (1.2 mg per mouse for one day); (**F**) MAAs-H group, local treatment with MAAs (2.4 mg per mouse for one day).

HE staining and scanning electron microscopy of Institute of Cancer Research (ICR ) mice skin histological tissue was used to analyze the photoaging damage of mice in each group. The degree of injury to skin keratinocyte cells and collagen fiber is shown in Figure 5 and Figure 6. Compared to the NC group, MC mice had obviously different epidermal and dermal skin tissue pathology, with epidermal thickening, keratinization, and a fracture phenomenon of the collagen fibers as well as further complications such as collagenous fiber conglutination and uniform distribution. This suggested that an ideal skin photoaging model had been established.

However, vitamin C, which is commonly used in control groups of mice and is an effective antioxidant as assessed by pathology observations, was associated with a tendency for tissue damage migration [16]. Simultaneously, in the treatment group, the degree of skin tissue damage was obviously alleviated with increased dosages of MAAs, resulting in thinner stratum corneum, epidermis protected from hyperproliferation and a minor disruption in the dermis, as shown in Figure 5. The adhesion of skin collagenous fibers became less pronounced. Meanwhile, a tight arrangement of collagen bundles was observed and the relative integrity of cell and tissue structure was maintained, as shown in Figure 6. 

The experimental results indicate that the MAA compound extracted from *Porphyra yezoensis* could effectively relieve skin photoaging damage. This is in agreement with reports that an MAA compound could act effectively against UV damage [15,17].

### 2.4. Effect of MAAs on UV Irradiation-Induced Depletion of Endogenous Antioxidant Enzymes

**Figure 7:**
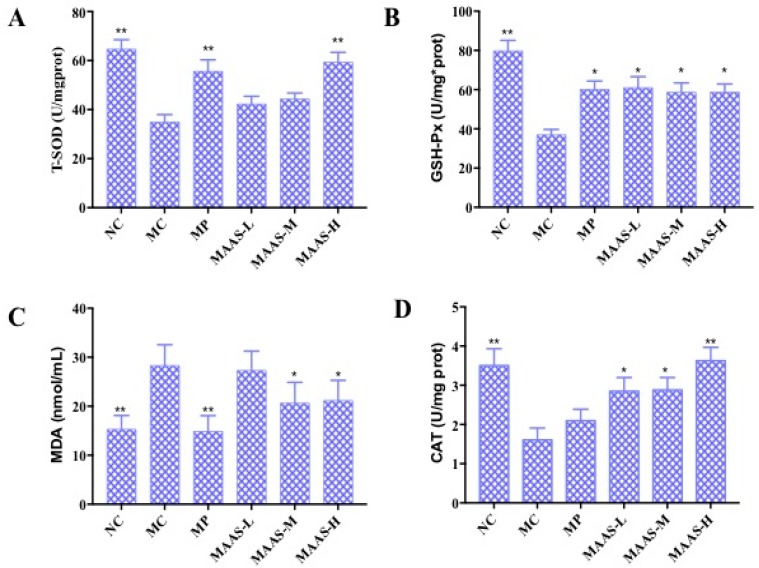
The effect of MAAs on antioxidant enzymes in mouse back skin tissue homogenate. (**A**) Effects of MAAs on the activity of total superoxide dismutase (T-SOD); (**B**) effects of MAAs on the activity of glutathione peroxidase (GSH-Px); (**C**) Effects of MAAs on the content of malondialdehyde (MDA); (**D**) effects of MAAs on the activity of catalase (CAT). Note: NC group was only treated with unhairing; the MC group received local administration of distilled water (120 μL/d) per mouse on the mouse back skin; the MP group received treatment with vitamin C solution (120 μL/d, concentration: 0.1 mg/mL); and the MAAs-L, MAAs-M, and MAAs-H groups were treated with MAAs solutions of 120 μL/d per mouse at respective concentrations of 5, 10, and 20 mg/mL. Compared with the model group, ** p < 0.01, * p < 0.05.

Ultraviolet radiation may cause oxidative damage to skin tissue and cell DNA as a result of the oxidative stress response due to the accumulation of ROS (including the superoxide anion radical, hydroxyl radical, and hydrogen peroxide) as reported [18]. The activity of endogenous antioxidant enzymes plays an important role in resisting UV irradiation-induced skin photoaging [19]. Figure 7 shows the activity of T-SOD, GSH-Px, and CAT, and the content of MDA in 10% skin tissue homogenate in all groups of mice that were subjected to the same dosage of ultraviolet radiation. Compared with the NC group, MC group mice that underwent UV radiation exhibited decreased T-SOD, GSH-Px, and CAT activity (*p* < 0.01), and a significantly higher level of MDA (*p* < 0.01). In contrast, MAA treatment groups were significantly and dose-dependently protected from T-SOD, GSH-Px, and CAT activity (*p* < 0.05, *p* < 0.01) and decreased levels of MDA (*p* < 0.05).

Of the three MAA treatment groups, the MAAs-H group had the greatest increase in endogenous antioxidant enzyme activity with increases in T-SOD, GSH-Px, and CAT activity in the skin tissue of 37.93%, 34.48%, and 22.22%, respectively compared with irradiated controls (MC). The content of MDA also decreased by 57.14%, as shown in Figure 7.

### 2.5. Effect of MAAs on Inflammatory Cytokine Levels in Photoaging Skin

**Figure 8:**
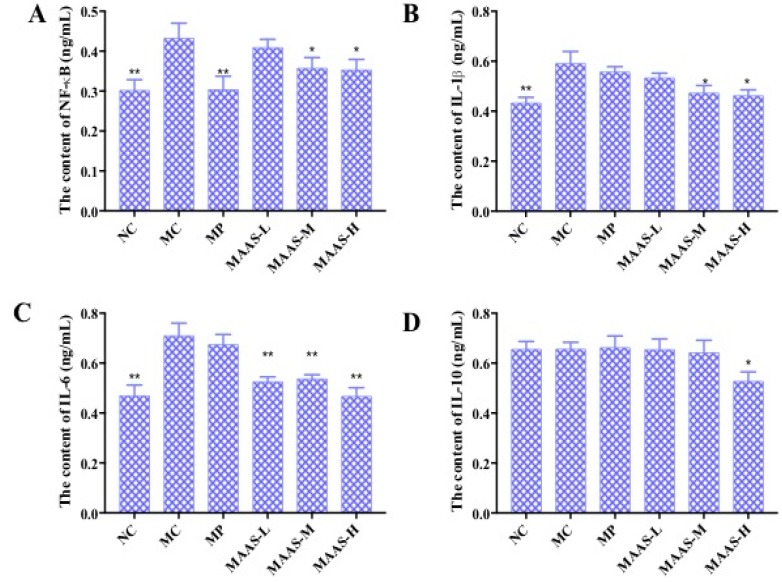
The effect of MAAs on inflammatory cytokine levels on the mouse back skin. (**A**) Effects of MAAs on the content of NF-κB; (**B**) Effects of MAAs on the content of IL-1β; (**C**) Effects of MAAs on the content of IL-6; (**D**) Effects of MAAs on the content of IL-10. Note: NC group was only treated with unhairing; the MC group received local administration of distilled water (120 μL/d) per mouse on the mouse back skin; the MP group received treatment with vitamin C solution (120 μL/d, concentration: 0.1 mg/mL); and the MAAs-L, MAAs-M, and MAAs-H groups were treated with MAAs solutions of 120 μL/d per mouse at respective concentrations of 5, 10, and 20 mg/mL. Compared with the model group, ** p < 0.01, * p < 0.05.

The levels of pro-inflammatory cytokines in mice skin tissue homogenate were measured by using an ELISA assay. As shown in Figure 8, in the MC group, the levels of NF-κB, IL-1β, and IL-6 in the skin tissue increased by 32.56%, 31.67%, and 39.44%, respectively, compared with the NC group (*p* < 0.01); however, there were no significant differences in the level of IL-10 compared with the NC group (*p* > 0.05), which may suggest that this factor is not associated with skin photoaging. This illustrates that ultraviolet irradiation could cause a significant rise in the level of pro-inflammatory cytokines. Compared with the MC group, the MAA treatment groups had significantly reduced levels of NF-κB, IL-1β, and IL-6 (*p* < 0.05 to 0.01), and these effects were dose-dependent. 

Of the three groups of MAA treatment, the MAAs-H showed the greatest inhibition of ultraviolet irradiation-induced pro-inflammatory cytokines, with reductions in NF-κB, IL-1β, and IL-6 activity of 20.9%, 28.33%, and 40.85%, respectively (*p* < 0.05 to 0.01), as shown in Figure 8.

### 2.6. Effect of MAAs on the mRNA Expression of Inflammatory Cytokines in Mouse Skin

**Figure 9:**
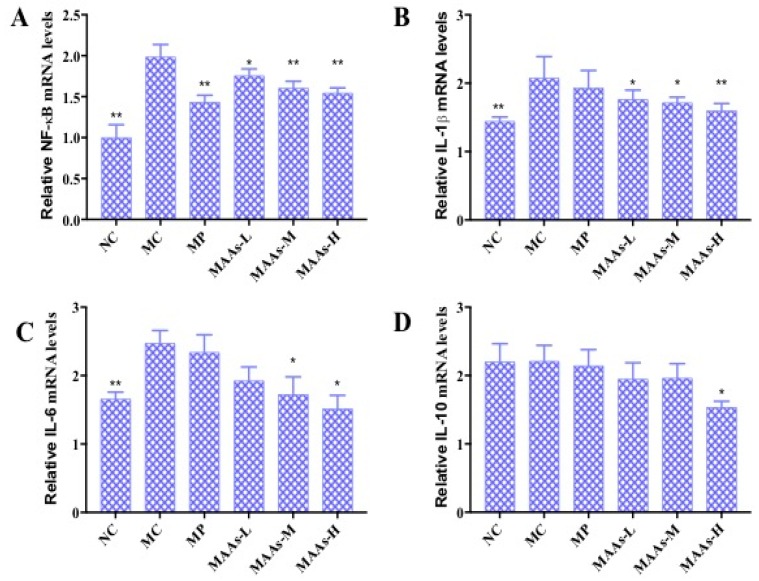
The effect of MAAs on the mRNA expression of inflammatory cytokine levels of mouse back skin. (**A**) Effects of MAAs on the mRNA expression of NF-κB; (**B**) Effects of MAAs on the mRNA expression of IL-1β; (**C**) Effects of MAAs on the mRNA expression of IL-6; (**D**) Effects of MAAs on the mRNA expression of IL-10. Note: NC group was only treated with unhairing; the MC group received local administration of distilled water (120 μL/d) per mouse on the mouse back skin; the MP group received treatment with vitamin C solution (120 μL/d, concentration: 0.1 mg/mL); and the MAAs-L, MAAs-M, and MAAs-H groups were treated with MAAs solutions of 120 μL/d per mouse at respective concentrations of 5, 10, and 20 mg/mL. Compared with the model group, ** p < 0.01, * p < 0.05.

NF-κB plays an important role in the expression of inflammatory cytokines, which is related to skin photoaging [9,19]. The mRNA expression levels of NF-κB, IL-1β, IL-6, and IL-10 were calculated and analyzed between different groups of mouse skin tissue. As shown in Figure 9, ultraviolet irradiation significantly increased the production of NF-κB, IL-1β, and IL-6 (*p* < 0.01), by 47.37%, 30.01%, and 34.54%, respectively. Compared with the MC group, the MAA treatment groups had significantly reduced mRNA expression of NF-κB, IL-1β, and IL-6 (*p* < 0.05 to 0.01), an effect that was shown to be dose-dependent. 

In this study, of the three MAA dosages, the MAAs-H group better inhibited the expression of NF-κB, IL-1β, and IL-6 mRNA with decreases in expression of 21.05%, 25.10%, and 42.13%, respectively (*p* < 0.05 to 0.01), as shown in Figure 9.

## 3. Discussion

Skin aging refers to cutaneous damage caused by endogenous metabolic and exogenous factors including sunshine, desert, and ultraviolet radiation [20]. Photoaging can occur as a result of chronic exposure of skin tissue to UV radiation, which causes skin histological pathological changes such as thickened, pigmentation, and infiltration of the dermis tissue with inflammatory cells [21]. The subsequent inflammatory response in skin tissue can lead to photoaging and even skin cancer [22]. For this study, we analyzed how MAA compounds protect against UV irradiation-induced photoaging and assessed the histomorphological changes and pathological damage. HE staining and SEM results were used to evaluate the protective efficiency of MAAs against UV radiation damage and the inflammatory reaction in skin tissue. The results of tissues and HE staining showed that MAAs can protect against UV irradiation-induced damage to skin, which includes histopathological lesions, such as skin erythema, edema, and collagen fiber breakage (Figure 5 and Figure 6). Furthermore, MAAs improved the disordered distribution of collagen fiber and improved the condition of the skin following photodamage. This illustrates that MAA compounds could effectively relieve UV irradiation-induced skin photodamage, depending on the strength of their ultraviolet absorption capacity. It can be used as a natural ultraviolet radiation absorbefacient and has important application value in sunscreen cosmetics and aesthetic medicine.

Meanwhile, the exposure of skin to UV radiation results in the production of a large amount of reactive oxygen species (ROS), which accumulate in the intracellular environment and disturb the redox balance. The resulting oxidative stress in skin tissue can lead to inflammation responses, cell death, and skin histopathological lesions, including cancer [22]. Simultaneously, this is associated with suppression of the activities of endogenous antioxidant enzymes, such as T-SOD, GSH-Px, CAT [9], and accumulation of the lipid peroxidation product MDA [19]. The significant impact of MAA compounds on resisting ultraviolet damage and increasing the activity of endogenous antioxidant enzymes was discovered and verified in the present research. Specifically, the content and activity of T-SOD, GSH-Px, and CAT in model skin tissue were significantly improved in a dose-dependent manner following MAA treatment (Figure 7A,B,D). In addition, MAA compounds inhibited the increase of the lipid peroxidation product MDA (Figure 7B). Thus, MAAs effectively restrain UV irradiation-induced skin photo-oxidative damage and lipid peroxidation, which can increase the activity of antioxidant enzymes and alleviate oxidative stress reaction. 

The ROS-mediated oxidative stress reaction causes an inflammatory response and suppresses the immune system during skin photoaging, during which the content and expression of proinflammatory cytokines continue to rise at different levels [23]. This experiment has shown that UV irradiation can upregulate the production of NF-κB, IL-1β, and IL-6, whereas the MAA compound can significantly inhibit this effect in a dose-dependent manner (Figure 8A–C). This indicates that MAAs could enhance immune protection against skin photoaging and alleviate the inflammation response caused by UV radiation by downregulating NF-κB, IL-1β, and IL-6.

On the other hand, the NF-κB signaling pathway plays an important role in the regulation of the mRNA and the protein expression of inflammatory cytokines [24]. We found that MAAs significantly inhibited the upregulation of NF-κB, IL-1β, and IL-6 mRNA expression in photoaging skin in a dose-dependent manner (Figure 9A–C). The results indicate that MAA compounds can inhibit expression of these compounds by regulating the NF-κB signaling pathway. Furthermore, MAAs were shown to effectively avoid immune-mediated photoaging damage, thus allowing the maintenance of normal collagen levels and cell structure integrity. In conclusion, MAA compounds could protect against UV irradiation-induced photoaging by enhancing the activity of antioxidant enzymes and inhibiting inflammation.

## 4. Materials and Methods

### 4.1. Materials

MAAs were obtained from *Porphyra yezoensis* and then separated and structurally confirmed. The commercial kits used to determine the contents of T-SOD, MDA, GSH-Px, and CAT were purchased from Jiancheng Inst (Nanjing, China). The commercial kits used to determine the contents of NF-κB, IL-1β, IL-6, and IL-10 were purchased from R&D Inst. (Minneapolis, MN, USA). The primers targeting mRNAs, including NF-κB, IL-1β, IL-6, and IL-10 were designed and synthesized by Shanghai Biotechnology Co., Ltd., Shanghai, China. Other chemicals and reagents used for this experiment were of analytical grade and are commercially available.

Male ICR mice (4 to 6 weeks) were purchased from the Institute of Zoology, Academia Sinica Beijing (Beijing, China). All animal procedures for this research conformed to the rules of international moral principles and the Guidelines for the Care and Use of Laboratory Animals [25]. Care and treatment of the animals were in accordance with the standard laboratory animal protocols approved by the Animal ethics Committee (the College of Food Science and Engineering, Ocean University of China Animal Experimental Ethics Committee, Qing Dao, China; Approval Date : March 7, 2016).

### 4.2. Preparation and Analysis of Mycosporine-Like Amino Acids

MAA compounds from Porphyra yezoensis were extracted by 15% ethanol aqueous solution. with ultrasonic for 2 hours and purified using SA-2 cation exchange resin, which were applied to analyze further the anti-ultraviolet radiation mechanism. The major bioactive components were analyzed using HPLC-UV-MS and concentration were calculated based on the molar absorptivity of prophyra-334 and shionrine, as previously described [12,26]. MAA compounds were characterized by contrasting the molar absorption coefficient and the maximum ultraviolet absorption spectrum at 200–400 nm with values presented in related literature [15]. The chemical structures and types of mycosporine-like amino acids were analyzed and identified by a Q-TOF high-resolution mass spectrometer equipped with the capability to perform electrospray ionization (ESI). The following parameters were used: SB-C18 chromatographic column, 20% methanol solution as eluent, 0.5 mL/min, 334 nm detection, and execution of the positive ionization mode from 100 to 1000 *m/z* for MS and 60 to 360 *m/z* for MS/MS. Then the samples were prepared, identified, and stored at –20 ℃ until use.

### 4.3. MTT Cytotoxicity Assay

L929 Fibroblast cells (obtained from Sangon Biotech (Co., Ltd., Shanghai, China) were used as the cell model to measure the toxicity of MAA compounds extracted from *porphyra yezoensis* as part of 4.2 by MTT assay (Sigma, St. Louis, MO, USA). The cultured cells that had been incubated in a 96-well plate (1 × 10 ^5^ initial cell density/well) at 100 μL per well were maintained at 37 ℃ in a humidified 5% CO_2_ incubator for 24 h, and then 10 μL was added to the test substances for model groups (the MAAs water solution concentrations were 0.5%, 1.0%, and 2.0% respectively). Dulbecco's modified eagle medium (DMEM) was only added to the control group for 12 h. Fifty microliters of MTT reagent (dilution of 1:5) was then added to L929 cells directly for 4 h at 37 ℃. The supernatant was removed, followed by the addition of DMSO (150 μL per well) to dissolve the formazan completely. The plate was then shaken, and the absorbance values of the cells were measured at 570 nm.

### 4.4. Experimental Groups

The experiment was performed after all animals had acclimatized for 1 week. They were treated with 6% sodium sulphide-ethanol solution for depilatory unhairing of the back over an area of 2 × 3 cm. It should be noted that the NC group was only treated with unhairing; the MC group received local administration of distilled water (120 μL/d) per mouse on the mouse back skin; the MP group received treatment with vitamin C solution (120 μL/d, concentration: 0.1 mg/mL); and the MAAs-L, MAAs-M, and MAAs-H groups were treated with MAAs solutions from part of 4.2 of 120 μL/d per mouse at respective concentrations of 5, 10, and 20 mg/mL. The solutions were absorbed for 30 min percutaneously, and then all groups were placed under specific conditions involving a combination of ultraviolet irradiations. The UV-induced pathological damage in skin tissues was observed daily after the irradiation experiment. 

### 4.5. Establishment of the UV Irradiation-Induced Skin Photoaging Model

The objective of this experiment was to establish an animal model that, using complex ultraviolet, could be used to research the mechanism by which MAAs regulate tissue antioxidant enzyme levels and the expression of inflammatory factors, thereby protecting against UV irradiation-induced photoaging. The following methods were used for the irradiation of unhaired mouse skin, as in our previous report [12]: One 40 W UVB (UVB-313; wavelength range: 280–320 nm) and two 40 W UVA (UVA-340; wavelength range: 320–400 nm) lighting tubes (length and diameter of the light tube: 1200 × 38 mm) were used for simulating the ultraviolet light source. The distance from complex ultraviolet light source to the backs of the mice was 30 cm. Meanwhile, a radiometer was used to measure the practical ultraviolet intensity. With these conditions, the average UV radiation intensities were UVA 2.91 mw/cm^2^ and UVB 18.07 mw/cm^2^, which total radiation dose were radiation intensities and multiply by the time (irradiation duration was calculated by minimal erythemal dose). In the experiment, all mouse groups received identical doses of ultraviolet light as the distance between the model mice and the UV tubes was adjusted every five minutes. For this modeling process, the damage accumulation method was used to track the appropriate UV irradiation dosing as follows: 0.5 MED (minimal erythemal dose) for the first week, and then the UV irradiation intensity was increased per week up to 4 MED. Ultimately, all groups of mice received radiation dosages as follows: UVA: 20.81 J/cm^2^, UVB: 0.47 J/cm^2^. The irradiation experiment lasted for about one month.

All groups of mice were sacrificed after the last UV irradiation experiment, and dorsal skins were collected so that the level of antioxidant enzymes and inhibition of inflammation in the skin tissue could be further analyzed.

### 4.6. Tissue Morphology and Histopathological Analysis 

Mouse dorsal skin samples were selected from different groups and sliced into one-quarter-inch strips and immersed in 10% buffered formalin for 72 h for tissue fixation. Afterwards, skin tissue was embedded with paraffin wax using the routine method, and then it was cut into slices. The pathological changes of skin tissue were observed with an optical light microscope after HE staining at 40× magnification.

### 4.7. Tissue Histopathological Analysis by Scanning Electron Microscope (SEM)

In other skin samples, all tissue materials were fixed with 2.5% glutaraldehyde for 7 days and used to analyze the extent of damage done to the local skin tissue and collagen fibers with a scanning electron microscope (SEM) [12]. In addition, all samples were treated by desiccation and gold spray to characterize the microstructure of the dermis slice tissue and collagenous fiber using a scanning electron microscope. (Olympus Optical Co., Ltd., Tokyo, Japan) at 1000× magnification.

### 4.8. The Effect of MAAs on Endogenous Antioxidant Enzymes

In accordance with the antioxidant activity assay directions, 10% skin tissue homogenate was prepared with phosphate buffered saline (1:9 w/v) and stored at −20 °C. The total protein content was measured using protein quantitative kit, and then the SOD, MDA, GSH-Px, and CAT concentrations in the mice skin tissue homogenate were measured and calculated using a reagent kit (NanJing JianCheng Bio Inst, Nanjing, China).

### 4.9. Effect of MAAs on the Expression of Inflammatory Cytokines 

The protein expressions of NF-κB, IL-1β, IL-6, and IL-10 in the skin homogenates were measured and calculated using ELISA kit (R&D, Minneapolis, MN, USA) assays, which were based on the total protein contents of skin tissue homogenates, as previously described [12,15]. 

### 4.10. RNA Isolation and Quantitative Real-Time PCR

A sample of every skin tissue was ground to a fine powder and the tissue total RNA was extracted using Trizol reagent (Invitrogen, Carlsbad, CA, USA). Then, the mRNA expressions of NF-κB, IL-1β, IL-6, and IL-10 was determined and analyzed following the instructions of the manufacturer [11]. Agarose gel electrophoresis was used to detect the integrity of the total RNA, and the concentration and purity of skin tissue RNA was determined using a Nanodrop ND-2000 (Thermo Fisher Scientific, Wilmington, MA, USA) based on conjoint analysis. 

Using reverse transcription PCR, the RNA extracted from skin tissue was reverse-transcribed to cDNA with M-MLV and random primers (Biotech Technology, Ltd., Shanghai, China). A 25 μL reaction mixture containing SYBR Green Master Mix (Promega Co., Ltd., Madison, WI, USA) and designed specific primer pairs, the prime sequences of which are listed in Table 1, was defined using quantitative real-time PCR (qRCR) reaction systems with the Line-Gene 9600 plus Detection System. The cycling conditions were as follows: 95 °C for 10 min, 40 cycles of 95 °C for 15 s, 58 °C for 20 s, and 72 °C 30 s [7,27]. The GADPH served as a housekeeping gene, and the mRNA levels of specific genes were normalized to GADPH. The relative mRNA expressions of NF-κB, IL-1β, IL-6, and IL-10 were calculated using the relative quantification (_ΔΔ_Ct) method following Livak [27]. 

### 4.11. Statistical ANALYSIS

All experimental data were analyzed by analysis of variance (ANOVA) with SPSS software (SPSS, Version 17.0, IBM Inc. Armonk, NY, USA). All statistical data are presented as the mean ± standard deviation (*n* = 10). Statistically significance was defined as *p* < 0.05.

## 5. Conclusions

This research objective of this study was to explore how MAAs extracted from *Porphyra yezoensis* protect against UV irradiation-induced photoaging by adjusting the activities of endogenous antioxidant enzymes and inhibiting inflammation. The results indicate that MAAs can effectively alleviate skin photo-oxidative damage, protect skin tissue, resist UV aging, and sustain the integrity of the structure of collagen fibers and cells. The MAA-related mechanisms of anti-photoaging were shown to be associated with the inhibition of endogenous antioxidant enzyme down-regulation and the suppression of inflammatory cytokine up-regulation and mRNA expression through regulation of the NF-κB signaling pathway. As a result, MAA compounds have the potential to be used as active substances in anti-photoaging skin treatments and inflammation reactions.

## Figures and Tables

**Table 1:** The names and sequences of primers.

Primer Name	Primer Sequences
GADPH	F:5’-CGTGTTCCTACCCCCAATGA-3’
	R:5’-ATGTCATCATACTTGGCAGGTTTCT-3’
NF-κB	F:5’-CACTGAGGAGACCACCCAAG-3’
	R:5’-GTAAACGCCGAAGATGATGG-3’
IL-β	F:5’-CTCCATGAGCTTTGTACAAGG-3’
	R:5’-TGCTGATGTACCAGTTGGGG-3’
IL-6	F:5’-GTGGCTAAGGACCAAGACCA-3’
	R:5’-TTCCAAGAAACCATCTGGCTA-3’
IL-10	F:5’-TCCTTGGAAAACCTCGTTTG-3’
	R:5’-CTTCAATTGCTTCCCAAGGA-3’
